# Accuracy and suitability of eating disorder screening tools for binge eating disorder and bulimia nervosa in a primary care setting: a systematic review and narrative summary

**DOI:** 10.3399/BJGPO.2025.0149

**Published:** 2026-04-08

**Authors:** Stella Kozmér, Ruichen Yin, Joseph Evans, Alex Burns, Jane Smith

**Affiliations:** 1 University of Exeter Medical School, University of Exeter, Exeter, UK; 2 Department of Psychology, University of Exeter, Exeter, UK; 3 University of Sheffield Medical School, University of Sheffield, Sheffield, UK; 4 Three Spires Medical Practice, Truro, UK

**Keywords:** screening, eating disorders, mental health, bulimia nervosa, binge-eating disorder

## Abstract

**Background:**

Despite available screening tools for eating disorders (EDs), the accuracy and suitability of these in identifying binge eating disorder (BED) and bulimia nervosa (BN) in a primary care setting are undetermined, despite BED and BN being the most common EDs.

**Aim:**

To evaluate the accuracy and suitability of ED screening tools for BED and BN in a primary care setting.

**Design & setting:**

A systematic review with narrative synthesis in a primary care setting.

**Method:**

Six databases were searched, including MEDLINE, PsycInfo, and Embase. Two independent reviewers screened studies for inclusion. Studies were included that assessed the accuracy and/or suitability of screening tools for BED and BN in primary care. Quality was assessed using the Mixed Methods Appraisal Tool. A narrative summary was created after integrating the data using a convergent segregated approach.

**Results:**

Four studies met inclusion criteria. The included studies reported on Binge Eating Disorder Screener-7 (BEDS-7), Eating Disorder Examination Questionnaire (EDE-Q), and SCOFF (sick, control, one stone, fat, food) screening tools. No studies reported on the accuracy of screening tools for BED and suitability for BN. BEDS-7 and EDE-Q screening tools reported variations in their suitability in primary care. The main barriers to implementation in primary care were time constraints and a lack of trust in screening. SCOFF showed high sensitivity (97.88%–100%) for BN but had lower specificity (89.6%–94.4%), increasing false positives.

**Conclusion:**

ED screening tools face feasibility and accuracy concerns for BED and BN. Further research is needed to validate screening tools’ accuracy and suitability in a primary care setting for BED and BN in the general population.

## How this fits in

This research is highly relevant to general practice, where early identification of eating disorders (EDs) is crucial for early intervention. Given that there is an increase in prevalence of binge eating disorder (BED) and bulimia nervosa (BN), with primary care providers often serving as the first point of contact or presentation for individuals with BED and BN, understanding the accuracy and suitability of screening tools for these EDs within the primary care setting is essential. Key findings indicate that commonly used eating disorder screening tools show variable suitability in primary care, with limited evidence on accuracy for BED and concerns about false positives for BN despite high sensitivity in tools like SCOFF. These results highlight the need for further validation of screening tools in primary care and suggest that improvements in feasibility, clinician trust, and diagnostic precision are essential to enhance early identification and management in practice.

## Introduction

Eating disorders (EDs) are serious mental health conditions characterised by disturbed eating patterns, a focus on body image, and a preoccupation with food, weight, or shape.^1^ The most common EDs are binge-type EDs, such as bulimia nervosa (BN) and binge eating disorder (BED), affecting approximately 4.2% of the global population,^2,3^ with suggestions of a much higher real prevalence.^3^ Both BED and BN are characterised by recurrent binge episodes, during which individuals consume a large amount of food in a short period of time without the ability to control the behaviour.^1^ BN is further characterised by compensatory behaviours such as self-induced vomiting, excessive exercise, or laxative use to counteract the potential effects of the binge episode such as weight gain.^1^ While BN and anorexia nervosa (AN) share some common characteristics, such as a focus on weight, when looking at ED symptoms dimensionally instead of categorically, BED and BN are suggested to share clinical presentations more than AN and BN.^4^

Delayed identification of BED and BN could lead to reduced quality of life,^5^ with an increased risk of mortality and comorbidities such as obesity or depression,^6–9^ suboptimal use of the healthcare system, and presents an economic burden.^10–12^ Hence, early identification of BED and BN is crucial. However, poorly performing screening tools can lead to overdiagnosis and overmedicalisation.^13^ Despite primary care being the hub for early identification,^14^ detection rates of BED and BN in primary care remain low.^15^

Several screening tools have been developed to identify EDs, including BED and BN.^16^ The most commonly used screening tools, both in research and practice, are the Eating Disorder Examination Questionnaire (EDE-Q)^17^ and SCOFF (sick, control, one stone, fat, food).^18^ However, there is an inconsistency in the validity of screening tools. One systematic review reported that SCOFF had high sensitivity in young females with AN when compared with clinical interviews.^19^ When compared with EDE-Q as a benchmark, SCOFF had lower sensitivity and specificity than Eating Disorders Screen for Primary Care and Screen for Disordered Eating.^20^ However, the convergence between EDE-Q and clinical interviews was low to moderate, as it both overestimated and underestimated AN and BN.^21,22^ This questions the efficacy of EDE-Q being used as a comparator in earlier studies.

While there is some evidence for good sensitivity of these screening tools for detecting EDs,^23–25^ mainly AN, more evidence is needed to understand screening accuracy in other EDs, such as BED and BN, and the potential suitability of screening tools for key stakeholders within the primary care setting. Feltner *et al* conducted a systematic review that aimed to address the general accuracy of ED screening tools in a comprehensive review on primary care in the US.^26^ However, measures of suitability and accuracy of these tools within primary care for BED and BN were not reported. In addition, studies were limited to the US. Hence, building on Feltner *et al*’s work and the gap identified by the authors, this systematic review aimed to evaluate the accuracy and suitability of ED screening tools for BED and BN in a primary care setting.

## Method

### Protocol

The protocol for this review was prospectively registered with the PROSPERO database of systematic reviews (registration number: CRD42024595253). This systematic review was conducted in accordance with the Preferred Reporting Items for Systematic reviews and Meta-Analyses (PRISMA) statement.^27^

### Data sources and search strategy

The search was conducted in the following databases in August 2024: CINAHL Complete (EBSCO), Cochrane Central Register of Controlled Trials (CENTRAL), Embase, MEDLINE, PsycInfo, and Global Health (Ovid). Keyword search included ‘binge*’, ‘bulimi*’, ‘general practi*’, ‘primary healthcare’, ‘screening*’, and ‘questionnaire*’ (see Supplementary Information S1 for full search strategy). Owing to the difference in MeSH term availability across databases, some adaptations were made to ensure standardisation. Backwards and forward citation was implemented. Grey literature was considered for search; however, it was not included owing to the limited time and resources of this study, and was judged to be unlikely to meet the quality required for inclusion in this review.

### Selection criteria

Original qualitative, quantitative, and mixed-method research that reported on the accuracy and/or the suitability of ED screening tools for BED and BN in a primary care setting in peer-reviewed sources were included in this review (see Table 1 for definitions). No restriction was applied for language, year of publication, country, or ED screening tool. The primary care setting included professions such as GPs, dentists, community pharmacists, optometrists, nurse practitioners, and psychological wellbeing practitioners (PWPs), and patients of all ages with BED or BN. Studies with a true screening population sample were included. However, studies with low prevalence testing in primary care were also included to allow for a more generic understanding of the current situation. Studies using a diagnostic population were excluded. Studies focusing on secondary and tertiary care settings were excluded. Studies where non-primary care healthcare professionals (HCPs) were administering the screening tools were excluded. Studies reporting exclusively on AN, other specified feeding or eating disorders, or avoidant or restrictive food intake disorder were excluded.

**Table 1.** Definition of terms

Term	Definition
Accuracy	Any objective measurement of a tool’s ability to accurately detect BED or BN
Suitability	Views and opinions of healthcare practitioners (HCPs) or patients on the usage of screening tools within the primary care context and/or on BED or BN

BED = binge eating disorder. BN = bulimia nervosa.

### Study selection

First, title and abstracts were screened by two independent reviewers (JE and RY) using EndNote. Potentially relevant studies were subsequently retrieved and screened in full text (JE and RY). After full-text screening, potential articles were assessed for inclusion by a third and fourth independent reviewer (SK and JS). Disagreements were resolved via discussion.

### Data extraction

Data from the included studies were extracted on the study-level by JE and RY. Extracted data included title, authors, year and country of publication, design, diagnostic and screening tool used, recruitment setting, sample characteristics, and accuracy and suitability results.

### Quality assessment

The quality of the articles was assessed by RY, SK, and JS independently using the Mixed Methods Appraisal Tool (MMAT).^28^ The MMAT assesses quality using a set of questions depending on the study design. We identified the design of the studies included for data analysis and then chose the corresponding measure.

### Data analysis

Owing to the amount and type of studies included, a meta-analysis was neither appropriate nor feasible for data synthesis; hence, we deviated from our original protocol. Extracted data were synthesised using a convergent segregated approach as described by the Joanna Briggs Institute (JBI),^29^ and a narrative summary was created to describe the accuracy and suitability of ED screening tools reporting separately for BED and BN.

## Results

### Study selection

The search results identified 3101 records, of which 1422 were duplicated. Seventy studies were eligible for full-text screening, of which four studies were included in data analysis. See Figure 1 for detailed selection process and Supplementary Information S2 for list of excluded studies.

**Figure 1.**
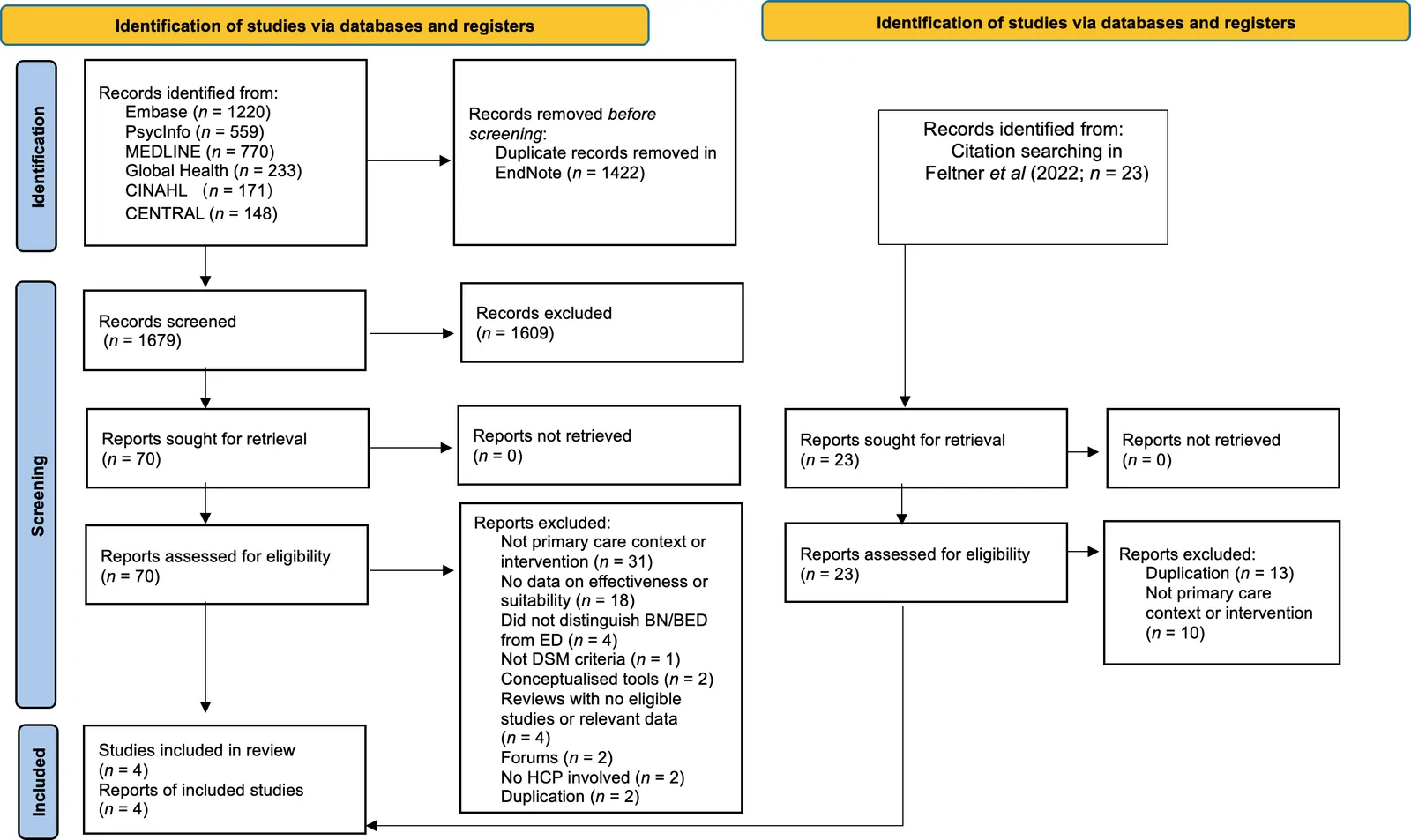
Preferred Reporting Items for Systematic reviews and Meta-Analyses (PRISMA) flow diagram. BED = binge eating disorder. BN = bulimia nervosa. DSM = *Diagnostic and Statistical Manual of Mental Disorders*. ED = eating disorder. HCP = healthcare professional.

### Overview of studies

The included studies were conducted in the UK,^30^ US,^31,32^ and Spain.^33^ Three studies reported on the accuracy of screening tools using sensitivity and specificity.^30,32,33^ Two studies reported on suitability.^31,32^ Two accuracy studies compared screening tools with clinical interviews. See Table 2 for further details.

**Table 2.** Characteristics of included studies

Lead author, year of publication	Location	Tool administrator	Population	Sex, %	Tool	Standard procedure	Condition	Data collection	Data analysis	COSMIN dimensions
McClure, 2020^31^	US	Nurse practitioners (*n* = 3) and registered nurse (*n* = 1)	Adolescent family clinic attendees without ED diagnosis (*n* = 7)	PCP sex unavailable Patients: M = 57.1 F = 42.9	EDE-Q	No comparison	BED	Screening: in-person survey during clinic visit; PCP views: pre- and post-implementation survey	Qualitative analysis, not further specified	Not reported
Herman *et al*, 2017^32^	US	N/A	Primary care physicians serving adults (*n* = 122) and psychiatrists (*n* = 123)	PCPs: M = 69.7 F = 30.3 Psychiatrist:M = 62.6 F = 37.4	BEDS-7	No comparison	BED	Online surveys of PCPs	Descriptive statistics	Not reported
Luck *et al*, 2002^30^	UK	Primary care professional	Sequential general practice attendees (*n* = 341)	F = 100	SCOFF	Clinical diagnostic interview based on DSM-IV (10–15 minutes)	BN, AN, EDNOS	Cross-sectional questionnaires (2 minutes)	Comparison with clinical interview diagnosis	Not reported
Garcia-Campayo *et al*, 2005^33^	Spain	Primary care professional	General practice attendees with a probable ED diagnosis assessed by GP (*n* = 203)	F = 100	Spanish SCOFF	Schedules for Clinical Assessment in Neuropsychiatry (SCAN)	BN, AN, EDNOS	Cross-sectional questionnaires (2 minutes)	Comparison with clinical interview diagnosis	Not reported

AN = anorexia nervosa. BED = binge eating disorder. BEDS-7 = Binge Eating Disorder Screener-7. BN = bulimia nervosa. COSMIN = COnsensus-based Standards for the selection of health Measurement INstruments. DSM-IV = *Diagnostic and Statistical Manual of Mental Disorders, fourth edition.* ED = eating disorder. EDE-Q = Eating Disorder Examination Questionnaire. EDNOS = Eating Disorder Not Otherwise Specified. F = female. M = male. N/A = not applicable. PCP = primary care provider. SCOFF = sick, control, one stone, fat, food.

### Risk of bias and quality

All studies were assessed to have medium quality and a medium risk of bias (see Table 3).

**Table 3.** Quality appraisal of included studies (mixed methods and quantitative descriptive)

**Mixed methods**					
**Lead author, year of publication**	**5.1. Is there an adequate rationale for using a mixed-methods design to address the research question?**	**5.2. Are the different components of the study effectively integrated to answer the research question?**	**5.3. Are the outputs of the integration of qualitative and quantitative components adequately interpreted?**	**5.4. Are divergences and inconsistencies between quantitative and qualitative results adequately addressed?**	**5.5. Do the different components of the study adhere to the quality criteria of each tradition of the methods involved?**
McClure, 2020^31^	Yes	Yes	Yes	Yes	Can’t tell
**Quantitative descriptive**					
**Lead author, year of publication**	**4.1. Is the sampling strategy relevant to address the research question?**	**4.2. Is the sample representative of the target population?**	**4.3. Are the measurements appropriate?**	**4.4. Is the risk of non-response bias low?**	**4.5. Is the statistical analysis appropriate to answer the research question?**
Luck *et al*, 2002^30^	Yes	Can’t tell	Yes	Can’t tell	Yes
Garcia-Campayo *et al*, 2005^33^	Yes	Can’t tell	Yes	Can’t tell	Yes
Herman *et al*, 2017^32^	Yes	No	Yes	Can’t tell	Yes

### Narrative synthesis

#### Binge eating disorder

Herman *et al* ^32^ explored the use of the BEDS-7, a condition-specific screening tool, among both HCPs in primary care and psychiatrists, with >75% of responders considering BEDS-7 to be either ‘very’ or ‘somewhat’ valuable and easy to use. According to HCPs, the primary reason for usage was to identify and initiate discussion on binge eating, while forgetfulness was the main reason for failing to use BEDS-7. Despite positive responses about usage, limited conclusions can be made about the suitability of BEDS-7 for BED. McClure^31^ reported similar findings on the usefulness of EDE-Q, a general ED screening tool, among HCPs in primary care in identifying BED risk factors in adolescents. However, the suitability of EDE-Q to the primary care setting was reported to be only adequate. While some HCPs acknowledged the potential positive benefits of using EDE-Q on adolescents’ health, others were unsure about the potential effects that EDE-Q integration into practice could have on patient care delivery. The main concerns about the implementation of screening into practice were stigma, lack of trust in screening, lack of trust in HCPs, and insufficient screening time. Only one HCP reported confidence in integrating EDE-Q without disrupting patient care. Overall, while a condition-specific tool is reported to be more useful and easier to use than a general ED screening tool, both have limitations in their implementation in practice. Hence, no firm conclusions can be made about the suitability of BEDS-7 and EDE-Q owing to limited data and a limited sample.

Regarding accuracy, no studies reported on specificity and sensitivity measures for BED. Two studies^30,33^ reported accuracy for Eating Disorder Not Otherwise Specified (EDNOS), which included BED in the *Diagnostic and Statistical Manual of Mental Disorders, fourth edition* (DSM-IV). However, owing to the separate categorisation for BED in the *DSM, fifth edition* (DSM-V), the statistical results did not allow for conclusions regarding the accuracy of screening for BED.

### Bulimia nervosa

No studies reported on the suitability of screening tools for BN or the primary care setting. Studies focusing on accuracy reported on sensitivity, specificity, and applicability in different patient populations. Both Garcia-Campayo *et al* ^33^ and Luck *et al* ^30^ reported high sensitivity of the SCOFF questionnaire (97.88% and 100%, respectively) for detecting BN, which might be owing to the population used in the study. However, differences between SCOFF versions are noted with Garcia-Campayo *et al* ^33^ reporting high specificity (94.4%) for the Spanish version, with a cut-off at >2 positive responses, while Luck *et al* ^30^ reported a lower specificity (89.6%) for the English SCOFF. However, these data did not separate the outcome for AN and BN; thus, we cannot make a strong conclusion for BN. Luck *et al* ^30^ reported a positive predictive value of 24.4% (95% confidence interval = 12.9 to 39.5) for SCOFF. Garcia-Campayo *et al* ^33^ argued that this predictive value could be owing to low ED prevalence in primary care. In two EDNOS cases, non-disclosure of symptoms resulted in missed cases. Hence, Luck *et al* ^30^ summarised that overidentification in this case could be acceptable, as patients who do not meet BN diagnostic criteria are harder to detect, and perhaps further questioning is needed after a positive screening result instead of automatic referral.

Despite both condition-specific and general ED screening tools being considered useful by HCPs for BED, feasibility concerns limited their perceived suitability in primary care. Similarly, with screening accuracy for BN, SCOFF demonstrated strong sensitivity but lower specificity, indicating a high risk of false positives. The lack of *DSM-V*-specific validation for BED screening and the limited heterogeneity in BN screening studies highlight the need for further research to refine tool applicability across diverse primary care populations.

## Discussion

### Summary

This systematic review aimed to explore the accuracy and suitability of ED screening tools on BED and BN in a primary care setting. Our synthesis highlights that screening tools for BED are perceived as useful by HCPs but face feasibility challenges owing to limited data on their accuracy. SCOFF showed high sensitivity and limited specificity for BN with no data on suitability. Overall, there was a limited amount of evidence available to draw strong conclusions. This could be owing to limited funding for ED research,^34^ debates around mental health screening,^35^ and the focus on secondary care in this field owing to prioritisation of AN and low-weight BN treatment.

### Strengths and limitations

To our knowledge, this is the first systematic review exploring the accuracy and suitability of ED screening tools for BED and BN in a primary care setting. A rigorous, systematic approach was used to address this, combining qualitative and quantitative literature, generating an in-depth understanding. However, the small number of eligible studies — 0ne of which being a dissertation — all had a medium risk of bias, lowering the reliability of conclusions. The evidence was not the most balanced, with only the accuracy or suitability of tools for each condition, not allowing a comparison of the tools’ performances for BN or BED. Furthermore, the differences in populations used to test the tools, combined with limited reporting of COSMIN (COnsensus-based Standards for the selection of health Measurement INstruments) dimensions, did not allow us to draw strong conclusions. However, it did highlight significant gaps in this field. A notable limitation was the reliance on studies using DSM-IV criteria for BN, which do not fully align with the current DSM-V diagnostic criteria. Future research should prioritise validating existing tools under the DSM-V criteria and explore their use in a primary care setting, to simulate how screening can be realistically implemented in HCPs’ routines. This could improve the understanding of the accuracy and suitability of screening tools for a wider population.

### Comparison with existing literature

Our findings suggest that even if a screening tool is accurate, its implementation into practice might be limited. This is similar to the literature on primary care delivery, which suggests that limited consultation times available for GPs pose a barrier to implementing additional screening.^36^ Furthermore, Johnston *et al* ^37^ reported HCP concerns about follow-up actions after a positive screen owing to potential differences in patient expectations around BED and BN management and the available pathways in their study exploring the feasibility of eating disorders in primary care. This was further supported by Bryant *et al*,^38^ whose systematic review recommended that a clear post-positive screen procedure needs to be in place for effective referral and treatment. Hence, further investigation is needed into the practical integration of BED and BN screening into primary care.

Findings from this review suggest that SCOFF might not be suitable for BED and BN in a primary care setting. This is in line with the literature, which suggests that SCOFF was developed without using a co-design framework,^18^ potentially making it less suitable for BED, BN, and primary care. However, not using co-design in the development of tools is not unheard of, as a systematic review reported limited input from individuals with lived experiences on clinical tool design, administration, or evaluation.^39^ Stakeholder engagement is crucial in the development process of screening tools as it can improve accuracy, suitability, engagement, and implementation into practice, as seen with the Patient Health Questionnaire.^40^ Hence, lived experience of BED and BN is important to be included in general ED screening tools to improve suitability and accuracy.^41^

Our findings highlight that SCOFF produced mixed results regarding its accuracy on BN identification in primary care. In clinical samples, SCOFF has been largely sensitive (69.6%–100%) and specific (73.6%–89.6%).^20,42,43^ However, this decreased in general population samples, where sensitivity ranged from 53.7%–77.4%, and specificity from 60.5%–93.5%,^44–46^ which is consistent with our findings. Kutz *et al* ^19^ commented that SCOFF was initially developed and validated in care-control studies in specific and homogeneous populations with a high prevalence of ED, such as young women with AN and BN. This suggests that SCOFF might not be effective for BED in primary care in a general population, especially considering the varying demographics of general practice populations, variations in clinical strategy, and governance between practices. Given that primary care settings are the first point of contact with health care and are the main hubs to identify BED and BN early, additional validation of SCOFF in more heterogeneous samples is needed.^19,38^

### Implications for research and practice

Our findings and the existing literature show that further research is needed to better understand the accuracy and suitability of ED screening tools for BED and BN in primary care. However, it is important to highlight that it is the psychometric properties of existing tools that need improvement, as suggested by Hay *et al*,^24^ rather than developing new tools. Based on the findings of this review, a summary of recommendations is presented by the authors in Table 4.

**Table 4.** Recommendations based on the review

Recommendation		Explanation
**Further understanding of currently available condition-specific and general ED screening tools**	**Validation studies**	The sensitivity and specificity of the BEDS-7 and EDE-Q, as well as the accuracy of the SCOFF for BED, should be evaluated in a primary care setting. Further empirical evidence is needed in the form of, for example, randomised controlled trials or large dataset studies to measure the health benefits of screening for BED and BN.
	**Suitability studies**	There is a need to assess the needs of stakeholders (primary care staff and people with lived experience) to address gaps in the suitability of existing screening tools for BED and BN in primary care.
**Exploring and addressing implementation barriers**		Further research should explore practical strategies to overcome barriers to screening implementation for eating disorders.
**Development and implementation of screening protocols**		Development of a clear post-positive screening protocol is recommended to tackle potential false positives and barriers to screening implementation.

BED = binge eating disorder. BEDS-7 = Binge Eating Disorder Screener-7. BN = bulimia nervosa. ED = eating disorder. EDE-Q = Eating Disorder Examination Questionnaire. SCOFF = sick, control, one stone, fat, food.

## Data

Materials and data used for the conduct of this research are available from the study authors on request.
